# Supplementary material for: The effects of spectral dimensionality reduction on hyperspectral pixel classification: A case study
Source: PLoS One. 2022 Jul 14;17(7):e0269174. doi: 10.1371/journal.pone.0269174 (PMC9282587; doi:10.1371/journal.pone.0269174)
Supplement: Attachment — Submitted filename: Response to Reviewers.pdf [file pone.0269174.s002.pdf]

Authors' Response to Reviews of

The Effects of Spectral Dimensionality Reduction on Hyperspectral Pixel Classification: A Case Study

Kiran Mantripragada, Phuong D. Dao, Yuhong He, and Faisal Z. Qureshi

---

EC: ***Editors' Comment***, **RC:** ***Reviewers' Comment***, AR: Authors' Response

1. Academic Editor

EC: ***The text of the manuscript is intelligible. The English language used must be corrected in several places***

AR: We have gone over the manuscript to improve the writing and fix the errors. Reviewer #1 provided us with a marked-up copy, which was very helpful. Thank you. We have addressed all the items identified in this document.

EC: ***To what extent would the results presented be generalizable to images of the same area made in different seasons or to images obtained in neighboring areas? Are simple correction ratios usable, possibly taking into account weather conditions?***

AR: This is an excellent question. The dimensionality reduction methods are (1) data-driven and (2) unsupervised. Consequently, there is a very good chance that it will be able to deal with imaging artifacts (presence of clouds, low-light conditions, atmospheric noise, etc.). The classification method is supervised and requires a set of labelled pixels for training. This suggests that if a classifier that is trained using artifact-free pixels is used as is to analyze pixels with artifacts, the performance of this classifier may suffer. On the other hand, it is perhaps possible to improve the performance of this classifier by training it on pixels exhibiting artifacts. We do agree that this question points to a highly promising direction for future work, and we plan to investigate this line of inquiry in the future.

EC: ***It would be interesting for authors to make the original images available in a file where interested parties can download and operate them to verify the results.***

AR: Yes, we plan to release the dataset, plus the source code, upon the publication of this work. We too feel strongly about this. It will not only increase the impact of this work, it will also allow others to explore other ideas using this dataset and code base. The data files and the source files will be available at `http://vclab.science.uoit.ca/datasets/ut-hsi301/`.

EC: ***The area mapped is represented by a star and does not provide sufficient clarity on the bounds and surface area covered.***

AR: Thank you for this suggestion. We now depict the region of interest using a bounding box. It better reflects the region from where the data used in this work was collected. Additionally, we plan to release the data files upon the publication of this work. These files are geo-referenced; therefore, it is also possible to pinpoint the area using these files.

EC: ***We note that you have provided funding information that is not currently declared in your Funding Statement. However, funding information should not appear in the Acknowledgments section or other areas of your manuscript. We will only publish funding information present in the Funding Statement***

section of the online submission form.

AR: Thank you for the guidelines. We removed the acknowledgements section and we will be providing information in the Funding Statement section of the submission form.

EC: ***According to the Section "Materials, Software and Code Sharing" of the submission guidelines.***

AR: We are also releasing the source-code used for the experiments. The files will be available at `https://github.com/kiranmantri/hsi-spectral-reduction`.

2. Reviewer #1

RC: ***to what extent would the results presented be generalizable to images of the same area made in different seasons or to images obtained in neighboring areas? Are simple correction ratios usable, possibly taking into account weather conditions?***

AR: Please see above.

RC: ***In order to make it easier for amateur readers to verify the processing of the original images, the authors should store the original images in an archive where interested parties can download and operate them.***

AR: Please see above.

RC: ***The English language used must be corrected in several places (English uk) either using the features of the MS Word editor, or, for example, the Grammarly free program, or another facility. I reported such minor errors in the reviewed manuscript attached to the review: PONE-D-22-02721_reviewer_rec.pdf***

AR: Thank you very much for thorough review and for the findings. We addressed the every one of the issues. Please see above in the answer to EC.

RC: ***It is recommended that you specify whether this symbol represents the space of vectors with real number components with 301 components. In other words, clearly define the mathematical symbol.***

AR: $\mathbf{x}_i \in \mathbb{R}^{301}$ denotes that $\mathbf{x}_i$ is a 301-dimensional vector of real numbers. Subscript i does not refer to an element of this vector, rather it denotes that vector $\mathbf{x}_i$ represents pixel i of the hyperspectral image. We have added this description to clarify this

RC: ***To increase the rigor, specify whether this symbol is the Euclidean norm in real vector space with 301 dimensions.***

AR: You are correct that it denotes the Euclidean norm in real vector space with 301 dimensions. We have added this description to the text.

3. Reviewer #2

RC: ***The paper is well structured and supported by good illustrations/graphs etc.***

AR: Thank you very much for your constructive comment.

RC: ***The area mapped is represented by a star and does not provide sufficient clarity on the bounds and surface***

area covered. If this can be changed for better readability and understanding.

AR: Thank you very much for the suggestion. We create new images to improve visualization. Please see above in the answer to EC.
